# Rapid Spread of Novel Infectious Bursal Disease Virus Variant (Genotype A2dB1b) in the Near East and Persian Gulf Regions: Molecular Surveillance and Phylodynamic Reconstruction

**DOI:** 10.1155/tbed/7950151

**Published:** 2026-01-23

**Authors:** Francesca Poletto, Safaa Alam, Abdallah Cherfane, Giovanni Franzo, Claudia Maria Tucciarone, Mattia Cecchinato, Matteo Legnardi

**Affiliations:** ^1^ Department of Animal Medicine, Production and Health, University of Padova, Legnaro (PD), Italy, unipd.it; ^2^ Near East Gulf and Sudan CEVA Animal Health LLC, Beirut, Lebanon

## Abstract

Infectious bursal disease virus (IBDV) is one of the most impactful pathogens of poultry, with disease manifestations ranging from acute forms to subclinical but immunosuppressive infections. This heterogeneity, accompanied by a significant antigenic variability, is sustained by high mutation rates and frequent reassortments between the two genome segments, along with less frequent recombination events. In recent years, the proposal of several classification systems relying on phylogeny contributed to the characterization of several new IBDV genotypes, shedding light on an increasingly diverse epidemiological scenario. One of the most notable examples is the discovery of novel variant IBDVs (nvIBDVs, genotype A2dB1b), which, after emerging in China around 2015, rapidly spread across East and Southeast Asia. More recently, nvIBDVs were also reported in Egypt and Argentina, prompting concern due to their well‐established immunosuppressive potential and divergent antigenic features. The detection of A2dB1b strains in Egypt elicited a molecular survey to track their spread within the Middle East. From November 2023 to November 2024, diagnostic samples were collected from 138 flocks in 7 Near East and Persian Gulf countries. The analyses revealed that 55 of them (39.9%) were positive for field strains belonging to 3 genotypes, suggesting a high infectious pressure. Two genotypes, A3B1c and A6B1a, were already reported in the region, although they were found in additional areas. On the other hand, A2dB1b was identified for the first time in Jordan, Lebanon, and the United Arab Emirates, representing a large share of the field viruses detected in these countries. Phylodynamic analyses revealed that this swift spread may have been caused by separate introduction events from Egypt, East Asia, and even South America, highlighting the complexity of IBDV epidemiology. The obtained results will be crucial to better tackle IBDV in the region, guiding monitoring activities and raising awareness toward its proper control.

## 1. Introduction

Infectious bursal disease (IBD), also known as Gumboro disease, is one of the best‐established viral diseases of chickens. It typically manifests with non‐specific signs such as prostration and dehydration, along with more indicative lesions that include bursal atrophy and hemorrhages in the thighs and pectoral muscles. Nonetheless, clinical signs may vary widely based on the strain involved, ranging from acute disease with high mortality to subclinical forms whose impact is due to the resulting immunosuppression [1].

The etiological agent, infectious bursal disease virus (IBDV), belongs to the species *Avibirnavirus gumboroense*, genus *Avibirnavirus*, and family *Birnaviridae*. It is an enveloped virus with a double‐stranded RNA genome made of two segments, named A and B [2]. Two IBDV serotypes, 1 and 2, are recognized, but only the first is virulent. Nonetheless, further subdivisions are possible considering the high genetic variability of IBDV, which in turn might have significant repercussions on pathogenicity and antigenicity [3]. Such heterogeneity is sustained not only by the high mutation rate typical of RNA viruses but also by genetic reassortment [4]. Recombination events are also reported, albeit less frequently [5, 6].

In recent years, multiple classification systems based on phylogenetic criteria have been proposed [3, 7, 8], providing a standardized platform for strain characterization. Such approaches rely on sequencing the gene coding for the capsid protein (VP2), which contains major determinants of antigenicity and virulence [9, 10], and often the one encoding the RNA‐dependent RNA polymerase (VP1), whose changes are also known to affect pathogenicity [2]. Since the two genes are located in different segments, considering both also allows us to detect reassortment events. These approaches prompted the execution of many epidemiological studies across the world, which helped document the rise of novel IBDV types in different parts of the world [11–14].

In this context, the Middle East makes no exception: although very virulent IBDVs (vvIBDVs, genotype A3B2) were long thought to be predominant [15–17], a recent study highlighted that they only represented a geographically restricted minority of circulating field strains [18]. On the other hand, strains with a very virulent VP2 and a classical‐like VP1 (genotype A3B1) appear predominant across the Near East, with distinct IBDVs (dIBDVs, genotype A4B1) and ITA strains (genotype A6B1) being reported in Persian Gulf countries [3, 18, 19]. All these atypical genotypes were associated, either experimentally or empirically, with insidious IBD forms [18, 20, 21], causing damages mostly through immunosuppression and posing different diagnostic and control challenges compared to traditional vvIBDVs.

The Middle East epidemiological situation was then further complicated by the entry of another emerging genotype, as novel variant IBDVs (nvIBDVs, genotype A2dB1b) have been described in Egypt since 2023 [22, 23]. Following their emergence in China around 2015, nvIBDVs quickly disseminated across East and Southeast Asia [24–26] and were later signaled in Argentina as well [13]. Similar to the previously mentioned genotypes, nvIBDVs are responsible for subclinical infections with severe immunosuppression [27] and have attracted significant scientific attention due to their transboundary spread, capability of early bursal infection [22, 24, 28] and divergent antigenic features [27, 29].

Following the entry of nvIBDVs in the region, an urgent reassessment of the epidemiological situation was therefore needed. This study reports the results of IBDV monitoring activities in the months following the first detection of nvIBDVs in the Near East, providing useful data to track their dissemination and estimate their potential way of introduction.

## 2. Materials and Methods

### 2.1. Sampling

The study was conducted on diagnostic samples collected between November 2023 and November 2024 in Near East and Persian Gulf countries. Within this 1‐year period, samples were collected from 138 flocks located in Lebanon (58), Jordan (35), the United Arab Emirates (14), Oman (12), Kuwait (9), Iraq (6), and Qatar (4). According to the information provided, samples mostly came from broilers (124 flocks) but also from layers (11) and breeders (4), vaccinated with different vaccination types and protocols. The age at sampling ranged from 16 to 53 days (mean: 31.0 days).

From each flock, 8 individual bursae were collected and imprinted onto FTA cards (GE Healthcare UK Limited, Amersham, UK).

### 2.2. Sample Processing

Each bursal imprint was processed individually by cutting a 4 × 4 mm^2^ piece, placing it in a 1.5 mL tube with 1 × PBS solution, and vortexing it for 30 s. Nucleic acids were then extracted using a QIAcube HT workstation with the QIAamp 96 Virus QIAcube HT kit (QIAGEN, Venlo, the Netherlands). The eluted samples were stored at ‐ 80°C.

### 2.3. Molecular and Phylogenetic Analyses

Two RT‐PCRs targeting the VP2 and VP1 genes were performed using the SuperScript III One‐Step RT‐PCR System with Platinum *Taq* DNA Polymerase kit (Invitrogen, Waltham, MA, USA). Specifically, the primers 743–1 (5′‐GCCCAGAGTCTACACCAT‐3′) and 743–2 (5′‐CCCGGATTATGTCTTTGA‐3′) [30] were used to amplify a 743 nt long segment of the VP2 gene, whereas a 751 nt long portion of the VP1 gene was targeted with the primers B‐Univ‐F (5′‐AATGAGGAGTATGAGACCGA‐3′) [31] and VP1‐shortR (5′‐TGGAAACAAAAGCCCGCATG‐3′) [18]. All positive samples were subjected to Sanger sequencing, which was conducted at the Macrogen Milan Genome Center (Milan, Italy) using the two primer pairs used in the respective RT‐PCR. The obtained chromatograms were quality checked and appropriately trimmed using 4 Peaks (Nucleobytes B.V., Aalsmer, the Netherlands), then consensus sequences were generated with ChromasPro (Technelysium Pty Ltd, Helensvale, QLD, Australia).

A preliminary analysis was conducted to establish whether the obtained sequences belonged to vaccine or field strains. In the latter case, they were then subjected to further phylogenetic analyses. In case of multiple detections of identical field strains within the same flock, only one was considered to avoid duplicates.

Two datasets, one for the VP2 and the other for the VP1, were created by adding the obtained field sequences to reference strains representing all genogroups defined in the classification system proposed by Wang et al. [7] and further refined by Wang et al. [14]. Using the MEGA12 software [32], the sequences were aligned with the MUSCLE algorithm, and then phylogenetic trees were generated with the maximum likelihood method and adaptive bootstrapping (threshold = 5.0%). The most appropriate substitution model was selected according to the Bayesian Information Criterion (BIC) score. The resulting trees were finally edited with the Interactive Tree of Life (iTOL) online tool [33].

### 2.4. Phylodynamic Analysis

To understand the origin and spatial migration patterns of the A2dB1b genotype, two datasets were created by considering all the VP2 and VP1 nvIBDV sequences with known detection dates and locations available in GenBank at the time of the study (15 April 2025).

The strength of the temporal signal was preliminarily evaluated using TempEst [34]. Then, the Bayesian serial coalescent approach implemented in BEAST X v10.5 [35] was applied to estimate key population parameters, including the time to the most recent common ancestor (tMRCA), evolutionary rate, and viral population dynamics. For each dataset, the best‐fitting nucleotide substitution model was chosen according to the BIC score, calculated using JModelTest [36], and a discrete‐state phylogeographic analysis was performed, following the method described by Lemey et al. [37].

The molecular clock model and the migration model were selected by calculating the marginal likelihood for each combination using both path‐sampling and stepping‐stone methods, as suggested by Baele et al. [38]. Based on the marginal likelihoods, Bayes factors (BF) were calculated to compare and select the final models implemented in the analysis (Supporting Table S1, S2). Viral population size changes over time were reconstructed using the non‐parametric Bayesian Skygrid model, focusing on relative genetic diversity (effective population size multiplied by generation time; Ne × τ) [39].

Two independent Markov chain Monte Carlo (MCMC) runs of 200 million generations were conducted. Log and tree files were combined using LogCombiner after discarding 20% of samples as burn‐in. The results were analyzed in Tracer v1.7, and only runs with effective sample sizes (ESS) >200 and adequate convergence and mixing were considered valid. Parameter estimates were summarized using mean values and 95% highest posterior density (HPD) intervals. Maximum clade credibility (MCC) trees were generated and annotated using TreeAnnotator, included in the BEAST package.

## 3. Results and Discussion

Out of the 138 investigated flocks, 43 tested positive solely for IBDV field strains (31.2%). Vaccine strains, all showing classical features, were found in 37 flocks (26.8%), whereas coinfections between field and vaccine strains were found in 12 cases (8.7%). The remaining 46 flocks were negative (33.3%). Field strains were thus detected in 39.9% of the flocks, suggesting a high infectious pressure, in agreement with recent results obtained in the same geographic setting [18]. However, the epidemiological scenario has rapidly changed in terms of circulating field types: 30 flocks were infected by nvIBDVs (genotype A2dB1b), 23 by viruses exhibiting a very virulent‐like VP2 and a classical‐like VP1 (genotype A3B1c), and 3 by ITA strains (genotype A6B1a). A single flock was infected by both A2dB1b and A3B1c IBDVs. Infections were detected as early as 20 days of age for A3B1c and A2dB1b, with an average age equal to 31.5 days. The sequences obtained from field strains were deposited in GenBank under accession numbers PV539628 ‐ PV539733. An overview of the results is provided in Table 1.

**Table 1:** Results of IBDV analyses obtained at flock level in different countries, classified as follows: Field, if only field strains belonging to the same genotype were detected; Vaccine, if only vaccine strains were detected; Coinfection, if multiple strain types (either of field or vaccine origin) were detected; Negative, if all samples collected from the flock tested negative.

Country	Field			Vaccine	Coinfection	Negative	Total
	A2dB1b	A3B1c	A6B1a				
Iraq	—	2	—	2	1^a^	1	6
Jordan	13	—	—	7	4^b^, 1^c^	10	35
Kuwait	—	—	—	5	—	4	9
Lebanon	6	7	—	13	2^a^, 4^b^	26	58
Oman	—	1	—	8	—	3	12
Qatar	—	1	2	—	1^d^	—	4
UAE	2	8	—	2	—	2	14
Total	21	19	2	37	3^a^, 8^b^, 1^c^, 1^d^	46	138

^a^Coinfection between A3B1c and vaccine strains.

^b^Coinfection between A2dB1b and vaccine strains.

^c^Coinfection between A2dB1b and A3B1c strains.

^d^Coinfection between A6B1a and vaccine strains.

The detected A3B1c were phylogenetically related to strains previously reported in the region but also in Russia, Kazakhstan, and Italy, which led to the definition of the Italian‐Russian‐Middle Eastern clade [40]. Aside from their unexplained geographic distribution and pathogenic features, such strains raise many questions from a phylogenetic perspective: although the combination of a very virulent‐like VP2 and a classical‐like VP1 suggests a reassortant nature, no possible ancestors are known with similar characteristics within segment B, which are quite divergent from other viruses belonging to genogroup B1. Islam et al. [7] suggested that this clade might eventually evolve into a separate genotype, and this peculiarity seems to be confirmed in the present study, in which the Italian‐Russian‐Middle Eastern clade has been investigated using the classification by Wang et al. [8]. The reliance on this classification system, primarily justified by its enhanced resolution power when dealing with novel variant IBDVs, further highlighted the uniqueness of these strains: compared to the two subclades defined by Wang et al. [14] within genogroup B1, namely B1a and B1b (with the latter grouping nvIBDVs), the Italian‐Russian‐Middle Eastern clade formed a distinct monophyletic clade, which was thus named B1c. Of note, Bayraktar et al. [41] recently reported the presence of reassortant strains with a very virulent‐like VP2 and a classical‐like VP1 in Turkey as well; however, these strains are unrelated to the Italian‐Russian‐Middle Eastern clade and appear phylogenetically closer to the so‐called Northwestern European reassortants [40, 42], subsequently falling within A3B1a. In the near future, it will thus be interesting to monitor whether these viruses will further spread southward to other Middle Eastern countries.

**Figure 1:**
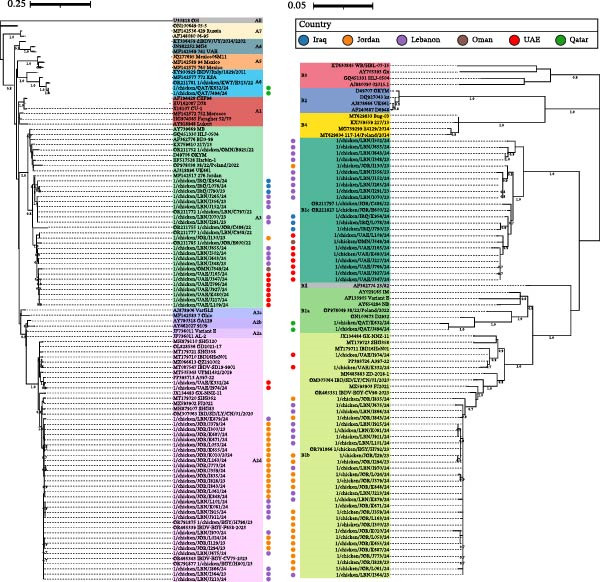
Phylogenetic trees of VP2 (left) and VP1 (right) field sequences, labeled with a circle and color‐coded according to the country of detection. Both trees were generated using the K2 + G substitution model [43], considering 429 and 510 nt long portions of the VP2 and VP1, respectively. Node support values are shown only if equal to or higher than 70.

The two detected A6B1a IBDVs were closely related to each other and clustered with ITA strains previously reported in the region, whereas two different subgroups could be defined among the sequenced A2dB1b sequences, one grouping those detected in the UAE and the other made of strains from Jordan and Lebanon (Figure 1).

**Figure 2:**
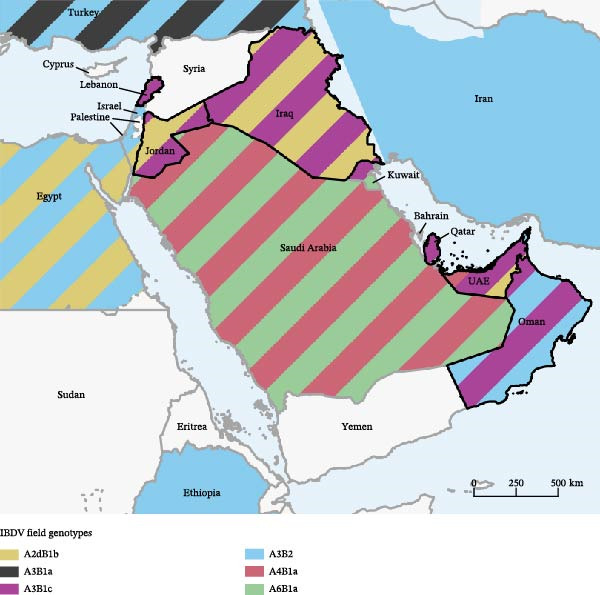
Distribution of field IBDV genotypes in countries investigated in the present study (black borders) and the surrounding region, according to newly detected strains and available publications and sequences. The map was prepared using QGIS 3.24 [44].

In terms of geographical distribution, A3B1c was found in Lebanon, Iraq, Oman, Qatar, and the UAE: while their presence in the Near East was already established [3, 18], this is the first report of this genotype in the latter three countries, expanding their known range to the Persian Gulf (Figure 2). On the other hand, A6B1 was only detected in Qatar: although no previous IBDV reports or sequences are available for this country, this finding is not particularly surprising, considering that this genotype has already been reported in Saudi Arabia [3] and Kuwait [18]. Nonetheless, the most interesting evidence is represented by the identification of nvIBDVs in different parts of the region: the first detection was in Jordan in November 2023, shortly followed by another in Lebanon in December 2023, whereas in the UAE they have been found since February 2024.

Continuous monitoring activities had been conducted prior to the beginning of this study, suggesting that these initial detections likely reflect the actual moment of A2dB1b introduction in the investigated countries, which appears to follow the earliest identifications in Egypt at the beginning of 2023 [22, 23]. Notably, the search for nvIBDVs within GenBank yielded an Israeli sequence from 2024 (chicken/ISR/8534‐bursa/2024, accession number PQ563200, direct submission). This finding demonstrates an even wider spread of this emerging genotype in the region. Additional sequences obtained in Iraq during 2023 were also retrieved, which were not clearly labeled as such in the related publication [45] but could be clearly classified within genotype A2dB1b.

Aside from the entry of A2dB1b in multiple countries, an even more worrying finding is the percentage of overall field strain detections that has been caused by these viruses: in the UAE, where they circulated for less time, nvIBDVs accounted for only 20% of field strains (2 out of 10); in Lebanon they represented 53% of them (10 out of 19); and in Jordan they were responsible for all the field IBDV detections (18 out of 18, although in one case A3B1c was also present in coinfection). These data suggest a swift and large‐scale penetration of this emerging genotype in new geographic and productive contexts, possibly at the expense of other circulating field IBDVs.

While these epidemiological findings appear indisputable and worthy of urgent dissemination, the present study is not exempt from limitations: notably, the reliance on convenience sampling hampered any inference about prevalence and, coupled with the limited number of samples received from some countries, might have contributed to the underreporting of additional field strain types. The assessment of vaccination efficacy was similarly hindered, even if such information was thoroughly collected: field detection rates, shown in Table 2, suggested a different effectiveness in preventing IBDV infections between vaccine types, which may be traced back to their different modes of action [46], but their uneven and sometimes insufficient representation prevented any robust conclusions on this subject. Moreover, the implemented study design failed to consider factors such as administration quality, which is also crucial for the resulting immunization, and the fact that vaccine types might not be evenly adopted in different countries, which, on the other hand, might suffer from different degrees of infectious pressure that might act as a confounding factor.

**Table 2:** Results of IBDV analyses obtained at the flock level, divided by vaccination regime, and classified as follows: Field, if only field strains were detected; Vaccine, if only vaccine strains were detected; Field‐vaccine coinfection, if both field and vaccine strains were detected; Negative, if all samples collected from the flock tested negative.

Vaccination	Field	Vaccine	Field‐vaccine coinfection	Negative	Total	Field detection rate (%)
Immune complex	28	30	8	39	105	34.2
Immune complex + live	—	3	1	2	6	16.7
Live	10	3	2	4	19	63.2
Vector	3	—	—	—	3	100
Vector + live	1	1	1	1	4	50
No vaccination	1	—	—	—	1	100
Total	43	37	12	46	135	40.7

Another limitation, albeit common to most studies on the subject, is the impossibility of detecting coinfections within individual samples using assays that amplify virtually any strain, whether of field or vaccine origin. Such coinfections are of great relevance for IBDV, as they represent an essential condition for the occurrence of reassortment events. Moreover, the competition with vaccine strains may exert a selective pressure on persisting field viruses, leading to antigenic drift [14]. Although the detection of coinfections would only be possible using multiple subtype‐specific tests or next‐generation sequencing approaches, this study reports several cases in which different strains (usually a field and a vaccine virus) were concurrently present within a single flock, clearly corroborating the possibility that such evolutionary mechanisms might be at play in the investigated productive context. Ideally, such situations should be prevented as much as possible by stopping field strain circulation, a goal that can only be pursued through prompt detection and implementation of effective vaccination protocols.

**Figure 3:**
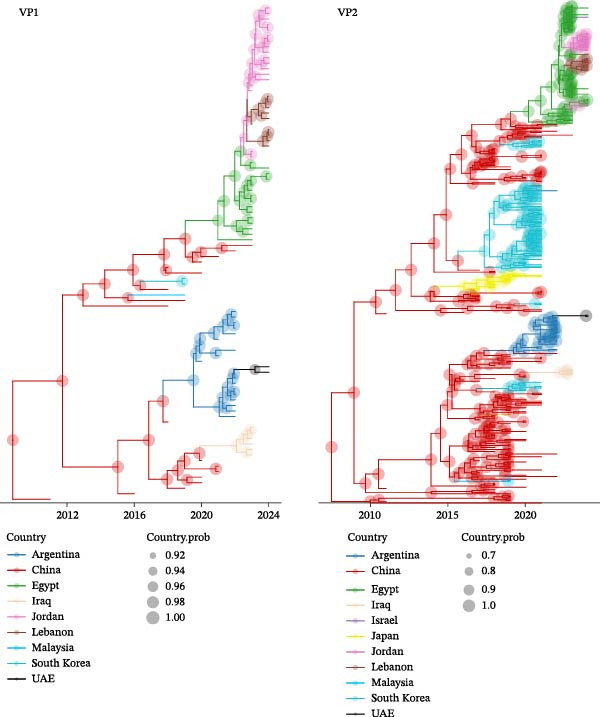
Maximum clade credibility (MCC) trees reconstructed based on all VP1 (left) and VP2 (right) A2dB1b sequences with known date and location of detection available at the time of the study. Branches were color‐coded based on the most probable ancestral location, while the node size is proportional to the respective posterior probability. The considered VP1 and VP2 portions were 384 and 510 nt long, respectively.

Another aim of this study was to understand the potential routes of A2dB1b introduction and dissemination in the region. To this purpose, 347 VP2 and 60 VP1 nvIBDV sequences were extracted from GenBank and used to perform a phylodynamic analysis, whose results are shown in Figure 3.

VP2 and VP1 analyses agreed on the predominant role of China (likely affected by the availability of many Chinese sequences) in the early dissemination of A2dB1b and in its intercontinental spread, whereas other Asian countries seem to support a mostly local circulation. However, the subsequent introduction in the Middle East appears to have occurred by multiple routes: the strains circulating in Jordan and Lebanon (with the addition of the aforementioned Israeli sequence) appear to have originated by the entry of Egyptian nvIBDVs; the source of those found in the UAE appears to be Argentina, which in turn showed ties with China; finally, the Iraqi strains were estimated to have an Asian origin without the mediation of other countries.

While puzzling at first glance, this scenario can be explained by considering factors such as geography and trade: while it is highly plausible that nvIBDVs might have spread from Egypt to nearby countries such as Jordan and then Lebanon, it is interesting to note that the UAE was the third biggest importer of poultry meat from both Argentina (10.7% of total exportations) and South America as a whole (9.26%) in 2023 [47]. Similarly, Saudi Arabia, for which no data on A2dB1b presence are currently available, was the second importer of poultry meat from Argentina (11.2%) and the fourth from South America (9.14%) [47] and could have also played a role in the entry of nvIBDVs. This claim is supported by the fact that this might not be the first instance an atypical IBDV genotype reached these countries from South America, as dIBDVs (A4B1), which are predominant in the continent [48], have been reported in both the UAE [3, 18] and Saudi Arabia [19]. Moreover, a previous phylodynamic investigation has proposed that the only dIBDV strain from the UAE available at the time might have originated from Brazil [49].

As for Iraq, it should be considered that, while sharing a border with Jordan, this country is the easternmost out of the investigated ones, and a westward introduction might thus have happened. Although recent information on the IBDV scenario in Central Asia is scarce, Zikibayeva et al. [50] reported the detection of a strain with an A2d VP2 (4_IBDV_2022, accession number PQ867576) in Kazakhstan in 2022, suggesting that this genotype might have been circulating in the region for some years. Unfortunately, this sequence had to be ultimately excluded from the phylodynamic analysis, as it was too short, and more epidemiological evidence would be needed to test this hypothesis.

## 4. Conclusions

The present study allowed us to promptly capture the entry of nvIBDVs in the Near East and Persian Gulf regions, seemingly due to multiple introduction events from different parts of the world. Despite being present for just a few months, these strains rapidly gained prominence, accounting for a substantial part of the overall field strain detections.

Aside from representing an interesting case study supporting the global nature of IBDV epidemiology, the obtained results should be interpreted as an urgent warning for the poultry industry in the Middle East. On top of the already circulating field genotypes, which have long exerted considerable infectious pressure, the emergence of the A2dB1b lineage poses additional challenges. The coexistence of strains with marked differences in terms of both pathogenicity and antigenicity and responsible for early infection demands careful planning of control measures, which, apart from reducing the disease burden, should be aimed at reducing field strain persistence and circulation. Although the optimization of biosecurity measures remains crucial, these objectives cannot be pursued without vaccination, whose efficacy should be maximized by addressing each of its aspects, from administration procedures to the choice of vaccination protocols granting early and homogenous immunization, up to the thorough assessment of their outcome. Control efforts should be coupled with steady and concerted monitoring, which will allow us to capture future epidemiological developments following the first phases of nvIBDV penetration, fill the identified knowledge gaps, and track the success of the implemented strategies.

## Funding

This research was supported by EU funding within the NextGeneration EU‐MUR PNRR Extended Partnership Initiative on Emerging Infectious Diseases (Project Number PE00000007, INF‐ACT) and by the University of Padova under Grant BIRD210528/21.

## Conflicts of Interest

The authors declare no conflicts of interest.

## Supporting Information

Additional supporting information can be found online in the Supporting Information section.

## Supporting information


**Supporting Information** Results of (log) marginal likelihood calculations performed using the Path Sampling (PS) and Stepping Stone (SS) methods, reported in log space. Estimations for the two genes are reported according to the clock and migration model combination, and the selected combination is highlighted in bold. Bayes Factor (BF) for every combination of clock and migration models, calculated with the PS and Stepping Stone (SS) methods for the two genes. The BF score describes the support for the model combination reported in the row (alternative model) against the one in the column (base model). The selected combination is highlighted in bold.

## Data Availability

The data that support the findings of this study are openly available in GenBank at https://www.ncbi.nlm.nih.gov/genbank/, reference number PV539628–PV539733.
