# Supplementary material for: Rapid Spread of Novel Infectious Bursal Disease Virus Variant (Genotype A2dB1b) in the Near East and Persian Gulf Regions: Molecular Surveillance and Phylodynamic Reconstruction
Source: Transbound Emerg Dis. 2026 Jan 23;2026:7950151. doi: 10.1155/tbed/7950151 (PMC12829470; doi:10.1155/tbed/7950151)
Supplement: Supplementary file 1 — Supporting Information Results of (log) marginal likelihood calculations performed using the Path Sampling (PS) and Stepping Stone (SS) methods, reported in log space. Estimations for the two genes are reported according to the clock and migration model combination, and the selected combination is highlighted in bold. Bayes Factor (BF) for every combination of clock and migration models, calculated with the PS and Stepping Stone (SS) methods for the two genes. The BF score describes the support for the model combination reported in the row (alternative model) against the one in the column (base model). The selected combination is highlighted in bold. [file TBED-2026-7950151-s001.docx]

Supplementary Table 1. Results of (log) marginal likelihood calculations performed using the Path Sampling (PS) and Stepping Stone (SS) methods, reported in log space. Estimations for the two genes are reported according to the clock and migration model combination, and the selected combination is highlighted in bold.

| Gene | Clock | Migration | PS | SS |
| --- | --- | --- | --- | --- |
| VP1 | Strict | Symmetrical | -2075,05 | -2075,48 |
|  | Relaxed | Symmetrical | -2074,56 | -2074,70 |
|  | Random | Symmetrical | -2072,78 | -2073,07 |
|  | **Strict** | **Asymmetrical** | -2065,97 | -2066,20 |
|  | Relaxed | Asymmetrical | -2077,00 | -2077,21 |
|  | Random | Asymmetrical | -2071,63 | -2071,91 |
| VP2 | Strict | Symmetrical | -6503,418 | -6509,53 |
|  | **Relaxed** | **Symmetrical** | -6477,90 | -6485,61 |
|  | Random | Symmetrical | -6487,60 | -6493,39 |
|  | Strict | Asymmetrical | -6491,56 | -6496,06 |
|  | Relaxed | Asymmetrical | -6532,98 | -6536,65 |
|  | Random | Asymmetrical | -6478,05 | -6489,48 |

Supplementary Table 2. Bayes Factor (BF) for every combination of clock and migration models, calculated with the Path Sampling (PS) and Stepping Stone (SS) methods for the two genes. The BF score describes the support for the model combination reported in the row (alternative model) against the one in the column (base model). The selected combination is highlighted in bold.

| Gene | Method |  | | | | | | |
| --- | --- | --- | --- | --- | --- | --- | --- | --- |
| VP1 | PS |  | Str-Sym | Rel-Sym | Ran-Sym | Stri-Asy | Rel-Asy | Ran-Asy |
|  |  | Str-Sym | - | 6,10E-01 | 1,03E-01 | 1,14E-04 | 7,03E+00 | 3,27E-02 |
|  |  | Rel-Sym | 1,64E+00 | - | 1,69E-01 | 1,87E-04 | 1,15E+01 | 5,36E-02 |
|  |  | Ran-Sym | 9,72E+00 | 5,93E+00 | - | 1,11E-03 | 6,83E+01 | 3,18E-01 |
|  |  | **Stri-Asy** | 8,74E+03 | 5,33E+03 | 9,00E+02 | - | 6,15E+04 | 2,86E+02 |
|  |  | Rel-Asy | 1,42E-01 | 8,68E-02 | 1,46E-02 | 1,63E-05 | - | 4,65E-03 |
|  |  | Ran-Asy | 3,06E+01 | 1,87E+01 | 3,15E+00 | 3,50E-03 | 2,15E+02 | - |
|  | SS |  | Str-Sym | Rel-Sym | Ran-Sym | Stri-Asy | Rel-Asy | Ran-Asy |
|  |  | Str-Sym | - | 4,60E-01 | 9,01E-02 | 9,40E-05 | 5,67E+00 | 2,83E-02 |
|  |  | Rel-Sym | 2,18E+00 | - | 1,96E-01 | 2,05E-04 | 1,23E+01 | 6,16E-02 |
|  |  | Ran-Sym | 1,11E+01 | 5,10E+00 | - | 1,04E-03 | 6,30E+01 | 3,14E-01 |
|  |  | **Stri-Asy** | 1,06E+04 | 4,89E+03 | 9,59E+02 | - | 6,04E+04 | 3,01E+02 |
|  |  | Rel-Asy | 1,76E-01 | 8,10E-02 | 1,59E-02 | 1,66E-05 | - | 4,99E-03 |
|  |  | Ran-Asy | 3,53E+01 | 1,62E+01 | 3,18E+00 | 3,32E-03 | 2,00E+02 | - |
| VP2 | PS |  | Str-Sym | Rel-Sym | Ran-Sym | Stri-Asy | Rel-Asy | Ran-Asy |
|  |  | Str-Sym | - | 8,24E-12 | 1,35E-07 | 7,10E-06 | 6,88E+12 | 9,61E-12 |
|  |  | **Rel-Sym** | 1,21E+11 | - | 1,64E+04 | 8,61E+05 | 8,34E+23 | 1,17E+00 |
|  |  | Ran-Sym | 7,39E+06 | 6,09E-05 | - | 5,25E+01 | 5,08E+19 | 7,11E-05 |
|  |  | Stri-Asy | 1,41E+05 | 1,16E-06 | 1,91E-02 | - | 9,69E+17 | 1,35E-06 |
|  |  | Rel-Asy | 1,45E-13 | 1,20E-24 | 1,97E-20 | 1,03E-18 | - | 1,40E-24 |
|  |  | Ran-Asy | 1,04E+11 | 8,57E-01 | 1,41E+04 | 7,38E+05 | 7,15E+23 | - |
|  | SS |  | Str-Sym | Rel-Sym | Ran-Sym | Stri-Asy | Rel-Asy | Ran-Asy |
|  |  | Str-Sym | - | 4,09E-11 | 9,82E-08 | 1,41E-06 | 5,97E+11 | 1,97E-09 |
|  |  | **Rel-Sym** | 2,45E+10 | - | 2,40E+03 | 3,46E+04 | 1,46E+22 | 4,81E+01 |
|  |  | Ran-Sym | 1,02E+07 | 4,16E-04 | - | 1,44E+01 | 6,08E+18 | 2,00E-02 |
|  |  | Stri-Asy | 7,08E+05 | 2,89E-05 | 6,95E-02 | - | 4,23E+17 | 1,39E-03 |
|  |  | Rel-Asy | 1,67E-12 | 6,84E-23 | 1,64E-19 | 2,37E-18 | - | 3,29E-21 |
|  |  | Ran-Asy | 5,08E+08 | 2,08E-02 | 4,99E+01 | 7,18E+02 | 3,04E+20 | - |
